# The Impact of Tobacco Use on Outcomes and Toxicity in a Predominantly Minority Population of Males with Prostate Cancer Receiving External Beam Radiation

**DOI:** 10.7759/cureus.1259

**Published:** 2017-05-18

**Authors:** Anna Lee, Meng S Shao, David Schwartz, Joseph Safdieh, Virginia W Osborn, David Schreiber

**Affiliations:** 1 Radiation Oncology, SUNY Downstate Medical Center & Veterans Affairs, New York Harbor Healthcare System; 2 Radiation Oncology, Kings County Hospital Center

**Keywords:** prostate cancer, radiation therapy, tobacco, smoking, race, african-american, toxicity

## Abstract

**Introduction:**

To investigate whether current or prior smoking history had any impact on prostate cancer outcomes and toxicity in our predominantly minority population of males receiving dose-escalated external beam radiation therapy (EBRT).

**Methods:**

Of the 500 patients treated with EBRT between 2003-2011, 444 had smoking histories recorded. Patients were classified as current smoker, former smoker, or never smoker. Biochemical failure-free survival (BFFS) and distant metastatic-free survival (DMFS) endpoints were analyzed. Multivariate Cox regression and multivariate logistic regression were used to assess whether smoking had an impact on outcomes and toxicity respectively.

**Results:**

There were 176 males (39.6%) classified as never smokers, 169 (38.1%) as prior smokers, and 99 (22.3%) as current smokers. The median follow-up was 76 months (range nine-146) and 61.9% of patients were African American. The eight-year BFFS for never smokers, prior smokers and current smokers was 73.6%, 80.2%, and 73.4% respectively, p=0.38. Similarly, the eight-year DMFS was 92.8%, 96.8%, and 95.3% respectively, p=0.54. On multivariate analysis, prior smoking (HR 0.72, p=0.19) and current smoking (HR 1.02, p=0.93) were not associated with increased biochemical failure. Similarly, smoking use was not associated with increased distant metastatic disease (hormone receptor (HR) 0.71, p=0.51 for prior smokers, HR 1.41, p=0.52 for current smokers). The presence of intermediate-risk disease (HR 2.87, p=0.002) was associated with an increased likelihood of biochemical failure. The high-risk disease was associated with both a higher risk of biochemical failure (HR 8.02, p <0.001) as well as distant metastatic disease (HR 17.61, p=0.01). On multivariate regression, prior or current smoking use was not associated with an increased likelihood of late grade two genitourinary or gastrointestinal toxicity.

**Conclusion:**

Current or prior smoking use was not associated with inferior outcomes or increased toxicity in this study comprising a predominantly minority population undergoing dose escalated radiation therapy for prostate cancer.

## Introduction

Over 480,000 Americans die every year from cigarette smoking and the costs associated with smoking-related illnesses approach 300 billion dollars per year [1]. It is well-known that cigarette smoking is directly linked to the increase in the incidence of head and neck, lung, stomach, pancreas, kidney, and bladder malignancies [2]. The association between smoking and the development of prostate cancer, however, is less clear [3-4].

The impact of cigarette smoking on outcomes is also not clear. Studies reporting on the impact of smoking on outcomes after radical prostatectomy or radiation therapy have often identified smoking as an independent predictor for biochemical recurrence and/or prostate-cancer-specific mortality [5-9]. However, other studies have conflicted with these findings [10- 11]. Additionally, two of these studies have suggested that smoking is also associated with increased toxicity from radiation therapy [7, 9].

The current retrospective study analyzed a cohort of United States Veterans, mostly consisting of a minority population, who received definitive dose escalated radiation therapy for prostate cancer. There are several reasons why having a high prevalence of African Americans may lead to unique findings. First, multiple studies have suggested that prostate cancer is biologically more aggressive and unique in African American males [12-17]. Therefore, the potential impact of smoking use may not necessarily translate in this more naturally aggressive cancer cohort. Furthermore, one of the proposed mechanisms accounting for the effect of smoking relates to the nicotine level, which has been shown to be higher in African American males than Caucasians [18]. Thus, we sought to compare outcomes and toxicity based on smoking status in our unique patient population. Informed consent statement was obtained for this study.

## Materials and methods

After the approval by our institutional review board, we reviewed the charts of all patients who were diagnosed with prostate cancer and were treated at the Brooklyn Veterans Hospital with external beam radiation to a dose of 7560cGy or higher, during 2003-2011. Smoking usage was identified based on the physician documentation at the time of consultation as well as the electronic medical record system, which often identified smoking use as part of the primary care physician note. Smoking usage was classified as current smokers, former smokers (quit before consultation or treatment started), or nonsmokers.

All patients were treated in the supine position for radiation therapy. Treatments were delivered via three-dimensional conformal radiation from 2003-2006. This typically involved a four-field box technique for the initial 4500cGy followed by a six-field oblique plan for subsequent boost fields. From late 2006 through 2009, intensity modulated radiation therapy (IMRT) without image guidance was utilized. Starting in 2010, image guidance was added via a daily megavoltage cone beam computed tomography scans matched either to the bony anatomy or to gold fiducial markers. The radiation fields varied based on the National Cancer Care Network (NCCN) risk group but typically included treatment of the pelvic lymph nodes for high-risk disease and prostate/seminal vesicles for those with intermediate- or low-risk disease.

Males who received androgen deprivation were treated with a luteinizing hormone agonist. The length of the androgen deprivation was based on physician discretion but was generally six months for intermediate-risk and two-three years for high-risk disease.

Upon completion of treatment, patients were followed every three-six months for five years, followed by yearly prostate-specific antigen (PSA) checks. The medical records of other clinics as well as other Veterans Hospitals, for those who moved away from our area, were also reviewed to determine any toxicity as well as to follow the PSA for patients who were lost to follow-up. Biochemical failure was defined using the Phoenix definition of PSA nadir + 2ng/mL.

Toxicities were graded using the National Cancer Institute Common Terminology Criteria for Adverse Events version 3.0. Grade one toxicity corresponded to minimal side effects, grade two toxicity corresponded to side effects requiring medications, grade three corresponded to side effects requiring minor procedures and grade four toxicity corresponded to medical admission due to life-threatening complications. Late (≥ three months after treatment) toxicity outcomes were compared between groups via the Chi Square test.

Patient characteristics were compared using Chi Square. Biochemical relapse-free survival (BFFS) and distant metastatic progression-free survival (DMPFS) were determined from the date of completion of radiation treatments. Their outcomes were analyzed using the Kaplan‑Meier method and compared using the log‑rank test. Multivariate Cox regression was used to analyze whether smoking had an impact on these endpoints. The variables were selected based on the likelihood of having an impact on outcomes and included age (continuous), race (White, Black, Hispanic), risk group (low-risk, intermediate-risk, high-risk), androgen deprivation (yes, no), radiation technique (three-dimensional conformal radiation therapy, intensity-modulated radiation therapy), and smoking history (never smoker, prior smoker, current smoker). 

Multivariate logistic regression was also used to analyze whether the smoking use was associated with increased toxicity when accounting for covariables. The covariables used were as noted above except age was a categorical variable for this analysis. The model was verified to have a non-significant p-value via the Hosmer and Lemeshow test. Statistical analysis was performed using SPSS version 23.0 [IBM Inc., Armonk, New York] and statistical significance was achieved with a p-value < 0.05.

## Results

There were 500 males treated with definitive radiation between 2004-2011, of which 444 had smoking histories recorded. There were 176 males (39.6%) classified as never smokers, 169 males (38.1%) as prior smokers, and 99 males (22.3%) as current smokers. The median follow- up was 76 months (interquartile range 52-106 months). The median age of all males was 70 years (interquartile range 62-75 years) and the median radiation dose was 7560cGy (range 7560-8100cGy). Details regarding patient characteristics by smoking category are available in Table 1.

**Table 1:** Patient characteristics by smoking category 3DCRT=three-dimensional conformal radiotherapy, IMRT=intensity modulated radiation therapy

	Never Smoker	Prior Smoker	Current Smoker	p-value
Age				<0.001
≤70	82 (46.6%)	74 (43.8%)	70 (70.7%)	
>70	94 (53.4%)	95 (56.2%)	29 (29.3%)	
Race				0.24
White	47 (26.7%)	52 (30.8%)	28 (28.3%)	
African American	108 (61.4%)	100 (59.2%)	67 (67.7%)	
Hispanic	21 (11.9%)	17 (10.1%)	4 (4.0%)	
Risk Group				0.12
Low	56 (31.8%)	41 (24.3%)	24 (24.2%)	
Intermediate	66 (37.5%)	80 (47.3%)	36 (36.4%)	
High	54 (30.7%)	48 (28.4%)	39 (39.4%)	
Androgen Deprivation				0.90
No	100 (56.8%)	97 (57.4%)	54 (54.5%)	
Yes	76 (43.2%)	72 (42.6%)	45 (45.5%)	
Radiation Technique				0.08
3DCRT	96 (54.5%)	88 (52.1%)	65 (65.7%)	
IMRT	80 (45.5%)	81 (47.9%)	34 (34.3%)	

**Figure 1:**
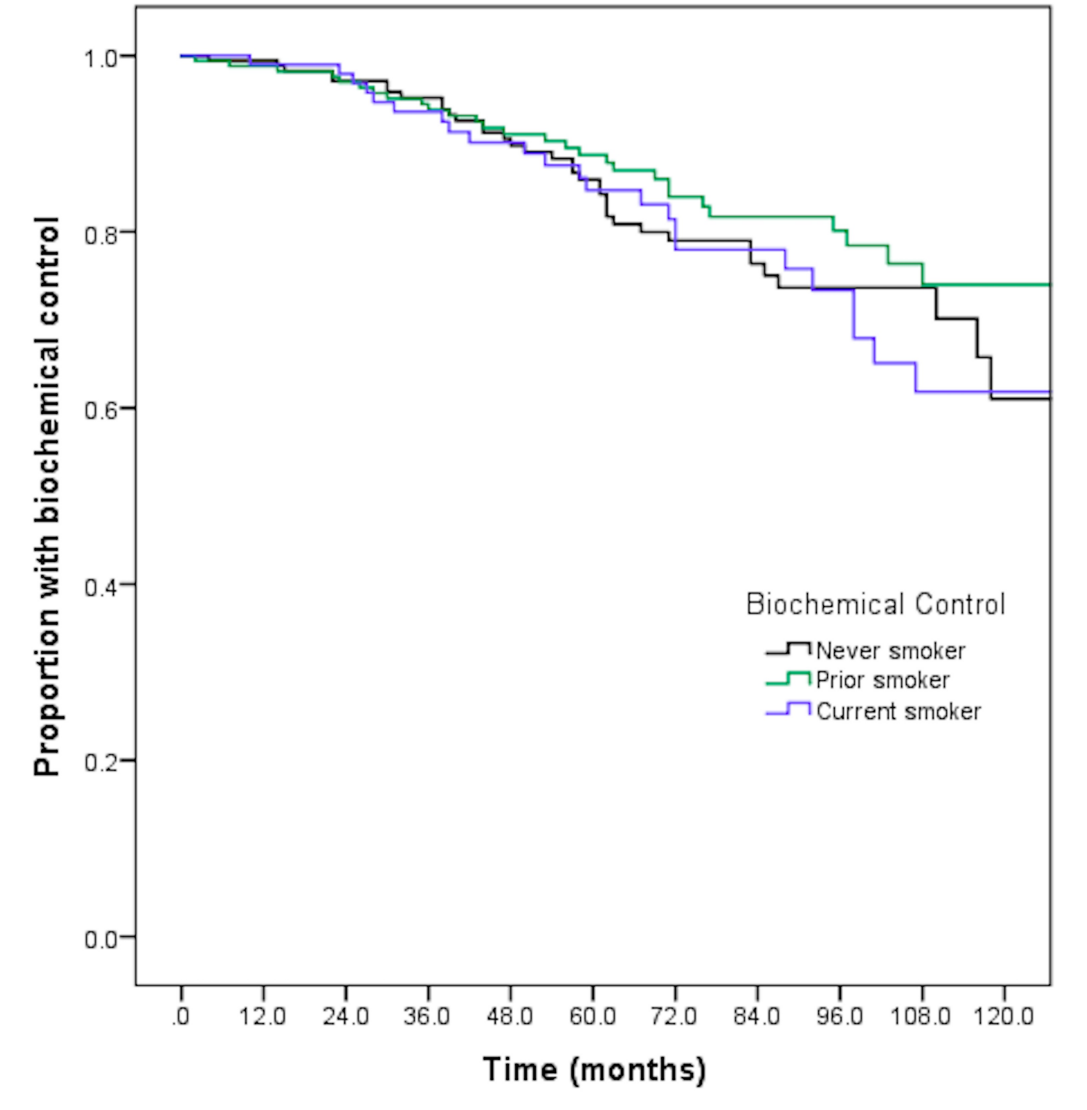
Eight-year biochemical failure-free survival by smoking status

The eight-year biochemical failure-free survival was 73.6% for the non-smokers, 80.2% for the former smokers, and 73.4% for the current smokers (p=0.38) (Figure 1). There were also no differences on pairwise comparison. On univariate analysis, there were no differences in biochemical control, based on smoking use (HR 0.75, p=0.26 for prior smoking and HR 1.07, p=0.80 for current smoking) or based on race (HR 1.06, p=0.82 for Black race and HR 0.52, p=0.18 for Hispanic race). On multivariate analysis, there remained no differences in biochemical control, based on smoking history or race. Prior smokers were noted to have a hazard ratio of 0.71 (95% CI 0.43-1.17, p=0.18) and current smokers were noted to have a hazard ratio of 1.06 (95% CI 0.61-1.83, p=0.84). Similarly, Black race had a hazard ratio of 1.02 (95% CI 0.63-1.65, p=0.93) and Hispanic race had a hazard ratio of 0.46 (95% CI 0.17-1.21, p=0.11). The addition of androgen deprivation was noted to be associated with an improvement in biochemical control (HR 0.40, 95% CI 0.23-0.72, p=0.002), while intermediate- or high-risk disease were associated with reduced biochemical control. Further details are available in Table 2. 

**Table 2:** Multivariate analysis for biochemical failure-free survival and distant metastatic-free survival HR=hazard ratio, 3DCRT=three-dimensional conformal radiotherapy, IMRT=intensity modulated radiation therapy

	Biochemical Control		Distant Control	
	HR (95% CI)	p-value	HR (95% CI)	p-value
Age (continuous)	1.03 (0.99-1.06)	0.06	1.05 (0.99-1.11)	0.07
Race				
White	1		1	
African American	1.02 (0.63-1.65)	0.93	0.66 (0.26-1.64)	0.37
Hispanic	0.46 (0.17-1.21)	0.11	0.54 (0.11-2.58)	0.44
Risk Group				
Low	1		1	
Intermediate	2.87 (1.45-5.68)	0.002	3.33 (0.38-29.12)	0.28
High	8.01 (3.54-18.13)	<0.001	17.61 (1.87-166.33)	0.01
Androgen deprivation				
No	1		1	
Yes	0.40 (0.23-0.72)	0.002	0.77 (0.24-2.49)	0.66
Radiation technique				
3DCRT	1		1	
IMRT	1.48 (0.92-2.38)	0.11	1.25 (0.47-3.33)	0.66
Smoking				
Never	1		1	
Prior	0.71 (0.43-1.17)	0.18	0.71 (0.25-2.02)	0.52
Current	1.06 (0.61-1.83)	0.84	1.45 (0.51-4.10)	0.49

**Figure 2:**
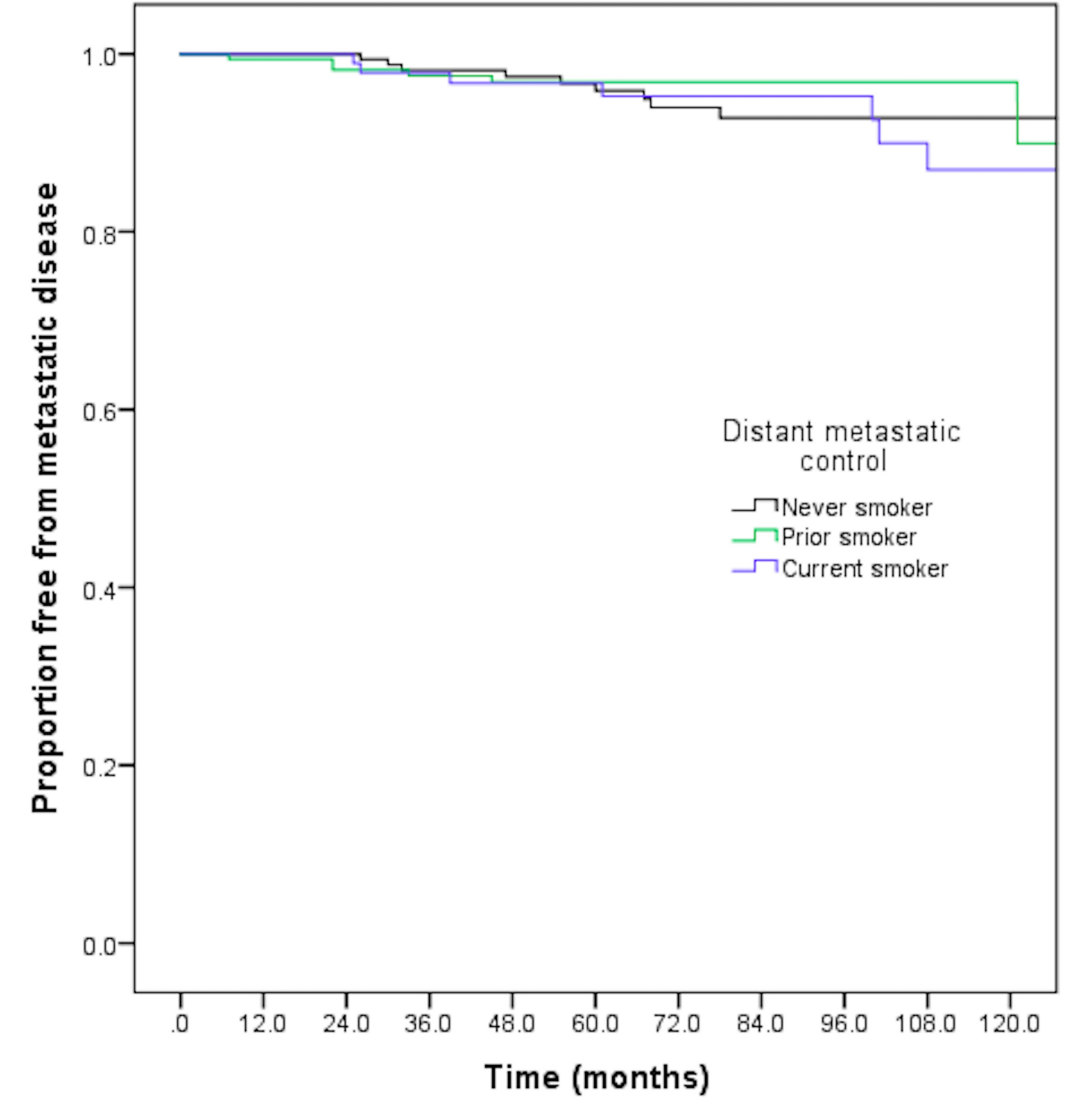
Eight-year distant metastatic-free survival by smoking status

The eight-year distant metastatic-free survival was 92.8% for non-smokers, 96.8% for prior smokers, and 95.3% for current smokers (p=0.54) (Figure 2). Similarly, there were no differences on pairwise comparison. On univariate analysis, neither prior smoking (HR 0.68, p=0.46) nor current smoking (HR 1.25, p=0.66) were associated with an increased likelihood of distant metastatic disease. Additionally, Black race (HR 0.57, p=0.20) and Hispanic race (HR0.62, p=0.54) also were not associated with an increased likelihood of metastatic disease. On multivariate analysis, only the presence of high-risk disease was associated with an increase in progression to metastatic disease (HR 17.61, 95% CI 1.87-166.34, p=0.01). Neither prior smoking history (HR 0.71, 95% CI 0.25-2.02, p=0.52) or current smoking (HR 1.45, 95% CI 0.51-4.10) were associated with an increase in distant metastatic disease. Further details are available in Table 2.

There were no significant differences between the smoking groups with regard to late toxicity. The late genitourinary toxicity was identified as being grade ≥ two for 23.9% of males with no smoking history, 27.8% of prior smokers and 17.2% of current smokers (p=0.14). Similarly, the late gastrointestinal grade ≥ two toxicity was 10.8% for no smokers, 13.6% for prior smokers, and 9.1% for current smokers (p=0.50). On multivariate logistic regression, age > 70 was associated with an increase in genitourinary toxicity. However, smoking use was not associated with any significant increase in late grade ≥ two genitourinary or gastrointestinal toxicity (Table 3).

**Table 3:** Multivariate logistic regression for late (≥ three months) genitourinary and gastrointestinal toxicity OR=odds ratio, 3DCRT=three-dimensional conformal radiotherapy, IMRT=intensity modulated radiation therapy, SV=seminal vesicle

	Genitourinary Toxicity		Gastrointestinal Toxicity	
	OR (95% CI)	p-value	OR (95% CI)	p-value
Age				
≤ 70	1		1	
> 70	1.61 (1.01-2.58)	0.04	1.74 (0.93-3.28)	0.09
Race				
White	1		1	
African American	0.96 (0.58-1.60)	0.88	1.27 (0.63-2.55)	0.51
Hispanic	1.20 (0.54-2.68)	0.65	0.87 (0.26-2.88)	0.81
Risk group				
Low	1		1	
Intermediate	1.32 (0.72-2.41)	0.36	0.84 (0.39-1.81)	0.66
High	0.61 (0.22-1.69)	0.34	0.36 (0.09-1.45)	0.15
Androgen deprivation				
No	1		1	
Yes	1.31 (0.71-2.41)	0.39	1.22 (0.54-2.77)	0.63
Radiation technique				
3DCRT	1		1	
IMRT	0.96 (0.52-1.75)	0.88	1.87 (0.81-4.33)	0.15
Radiation field				
Prostate/SV	1		1	
True pelvis	0.75 (0.36-1.56)	0.44	1.3p (0.47-3.60)	0.61
Whole pelvis	1.08 (.43-2.75)	0.87	1.59 (0.45-5.61)	0.48
Smoking				
Never	1		1	
Prior	1.18 (0.72-1.93)	0.51	1.26 (0.65-2.43)	0.50
Current	0.79 (0.41-1.51)	0.47	1.04 (0.44-2.45)	0.94

## Discussion

We found that current or prior smoking use during dose escalated radiotherapy for prostate cancer had no impact on outcomes or toxicity in our predominantly minority patient cohort of United States Veterans. Prior reports have been somewhat conflicting with respect to the effect of smoking on outcomes among prostate cancer patients, with most suggesting worse outcomes associated with smoking, and much fewer suggesting that smoking had no impact on outcomes. However, our patient population represents a unique cohort comprising of mostly minority Veterans, which differs from the patient populations in many of these studies.

Several studies have suggested that smoking is associated with worse outcomes. However, the careful review of these studies reveals that after adjusting for stage and grade at presentation, smoking may not be an independent predictor of worse outcomes. The largest study to date suggesting worse outcomes associated with smoking was reported by Steinberger, et al. [9]. In this study, they analyzed 2,156 males who received external beam radiation at Memorial Sloan Kettering Cancer Center. They found that 164 males (7.6%) were found to be current smokers, compared to 22% in the present study, and these males had a hazard ratio for biochemical recurrence of 1.40, p=0.02 and a hazard ratio for prostate-cancer-specific mortality of 2.25, p <0.001. The low overall smoking rate (7.6%) is much lower than the 25% reported in National survey data [19], which leaves open the possibility of selection or reporting bias. In addition, the race was not accounted for in their study.

In another study, the largest in the literature to date, by Kenfield, et al. [6] also reported on their observational cohort of 5,366 males with prostate cancer. They found that smokers were associated with higher biochemical recurrence and higher prostate-cancer-specific mortality. However, a closer look into the radiation related data reveals that there were no outcome differences in males who received radiation therapy, suggesting that it is not clear if smoking impacts prostate cancer radiotherapy outcomes. There were approximately 1,800 males who received radiation therapy, and of these, 95 (5.2%) were smokers. They reported that after adjusting for stage and grade, the hazard ratio for biochemical recurrence was 1.33 (95% CI 0.70-2.52) for patients who had radiation and the hazard ratio for prostate cancer-specific mortality was 2.07 (95% CI 0.60-7.08). Additionally, it should be noted that while the present study is much smaller overall, there were actually more smokers identified, as the smoking prevalence was only 5.2% in the aforementioned observational cohort. Finally, the impact of race was not assessed in the observational study.

Additional studies of smoking and prostate cancer outcomes after radiation therapy have suggested worse outcomes in smokers. However, similar to the observational study by Kenfield, et al., these data are no longer significant after adjusting for other covariables such as stage and grade. One such study of 633 males reported by Solanki, et al. [7] stated worse biochemical control with current smokers but this was no longer significant on multivariate analysis. A second, older study by Pickles, et al. [5] also reported worse outcomes amongst smokers in a cohort of 601 males but noted that smokers were more likely to present with younger age and more advanced T-stage, suggestive of systematic imbalances between the groups.

Finally, a large retrospective study by Taira, et al. reported on 2,057 males who underwent prostate brachytherapy, of which 15.7% were smokers [10]. They found that although smokers presented with more aggressive disease and had significantly worse overall survival (HR 2.599, p <0.001), there were no differences in biochemical control or cancer-specific survival. They postulated that smoking use was associated with more aggressive disease but a high-quality radiation treatment, in their particular study via prostate brachytherapy could overcome these inherent differences between smokers and non-smokers.

In total, the above studies suggest that smoking use is associated with more aggressive prostate cancer, but are not convincing that smoking influences outcomes for these patients after adjusting for treatment received and stage at diagnosis. Our present study adds to this mixed body of evidence and suggests that smoking may not negatively impact outcomes with radiation therapy. Our study is unique to prior investigations on two levels. First, our smoking rate was 22.9%, which was higher than all of the aforementioned reports, that ranged from 5.2%-19.6%, but is more consistent with prior National survey data [19]. In addition, while these reports did not include racial demographic information, we assume that African Americans were a minority population. This is in contrast to our study where African Americans comprised 61.9% of the population. This suggests that there may be a degree of underreporting in those studies relative to our database, likely due to the inherent racial and ethnic differences in the patient makeup of our patient cohort.

The impact of smoking use on toxicity has been assessed in two prior studies. Theoretically, smoking use may be associated with tissue hypoxia or changes in microvasculature that may affect tissue healing [20-21]. In the previously noted study by Solanki, et al., there were no differences in Grade three toxicity between smokers and non-smokers [7]. In an analysis of a large randomized trial assessing the impact of dose-escalated radiation therapy, Peeters, et al. reported that smoking was associated with a higher likelihood of dysuria requiring medications [22]. However, there were no other differences in toxicity between smokers and non-smokers. The present study is mostly in agreement with these prior analyses that there was not any apparent impact on toxicity associated with smoking use.

The limits of this study are inherent in its retrospective nature. These include lack of consistent details regarding the quantitative smoking use in pack-year units, as there may be effects present in a heavy smoker that were unmeasured due to the inclusion of many infrequent smokers in the current smoking group. Additionally, we do not have data identifying whether patients quit smoking during treatment or in between consultation and treatment initiation. Furthermore, we did not collect data on duration of smoking cessation. Smoking status is also a self-reported variable and susceptible to fabrication and bias. Finally, it is possible that our study of 444 patients was underpowered to detect the impact of smoking.

## Conclusions

Several prior studies have suggested that tobacco use is associated with worse outcomes and/or toxicity in males receiving radiation therapy for prostate cancer. Numerous reports have suggested that prostate cancer in African American males is a biologically unique disease compared to Caucasians. However, no previous study has assessed the impact of tobacco use in an African American population. In the present study comprising 444 males, of which 61.9% were African American, we found no association between tobacco use and prostate cancer outcomes or toxicity. Further studies of primarily African American males are needed to confirm our results.
